# Antibody Persistence in Young Children 5 Years after Vaccination with a Combined Haemophilus influenzae Type b-Neisseria meningitidis Serogroup C Conjugate Vaccine Coadministered with Diphtheria-Tetanus-Acellular Pertussis-Based and Pneumococcal Conjugate Vaccines

**DOI:** 10.1128/CVI.00057-16

**Published:** 2016-07-05

**Authors:** Juan Carlos Tejedor, Jerzy Brzostek, Ryszard Konior, Detlef Grunert, Devayani Kolhe, Yaela Baine, Marie Van Der Wielen

**Affiliations:** a Unidad Neonatal, Hospital Universitario de Móstoles, Madrid, Spain; b Pediatrics Department, Zespol Opieki Zdrowotnej w Debicy, Debica, Poland; c Neuroinfection and Pediatric Neurology, John Paul II Hospital, Cracow, Poland; d Praxis, Nordlingen, Germany; e GSK Vaccines, Bangalore, India; f GSK Vaccines, King of Prussia, Pennsylvania, USA; g GSK Vaccines, Wavre, Belgium; Duke University Medical Center

## Abstract

We evaluated antibody persistence in children up to 5 years after administration of a combined Haemophilus influenzae type b (Hib)-Neisseria meningitidis serogroup C (MenC)-tetanus toxoid (TT) conjugate vaccine coadministered with a pneumococcal conjugate vaccine. This is the follow-up study of a randomized trial (ClinicalTrials.gov registration no. NCT00334334/00463437) in which healthy children were vaccinated (primary vaccinations at 2, 4, and 6 months of age and booster vaccination at 11 to 18 months of age) with Hib-MenC-TT or a control MenC conjugate vaccine, coadministered with diphtheria-tetanus-acellular pertussis (DTPa)-based combination vaccines (DTPa/Hib for control groups) and a pneumococcal conjugate vaccine (10-valent pneumococcal nontypeable H. influenzae protein D conjugate vaccine [PHiD-CV] or 7-valent cross-reacting material 197 [CRM_197_] conjugate vaccine [7vCRM]). MenC antibody titers were measured with a serum bactericidal antibody (SBA) assay using rabbit complement (i.e., rabbit SBA [rSBA]), and antibodies against Hib polyribosylribitol phosphate (PRP) were measured with an enzyme-linked immunosorbent assay. Antibody persistence up to 5 years after booster vaccination is reported for 530 children ∼6 years of age. The percentages of children with seroprotective rSBA-MenC titers were between 24.2% and 40.1% in all groups approximately 5 years after booster vaccination. More than 98.5% of children in each group retained seroprotective anti-PRP concentrations. No vaccine-related serious adverse events and no events related to a lack of vaccine efficacy were reported. Approximately 5 years after booster vaccination, the majority of children retained seroprotective anti-PRP antibody concentrations. The percentage of children retaining seroprotective rSBA-MenC titers was low (≤40%), suggesting that a significant proportion of children may be unprotected against MenC disease. (This study has been registered at ClinicalTrials.gov under registration no. NCT00891176.)

## INTRODUCTION

Immunization of children with conjugate vaccines has proven to be a successful strategy to prevent infections caused by various encapsulated bacteria, such as Neisseria meningitidis and Haemophilus influenzae type b (Hib) (1), and has resulted in great decreases in the incidence of N. meningitidis serogroup C (MenC) and Hib disease in the world (2, 3). In addition to immune memory, persisting antibodies have been suggested to be a more appropriate correlate of long-term protection against disease (4). Circulating antibodies are particularly important for sustained protection against invasive MenC disease (5).

In Europe, MenC is the second most important cause of invasive meningococcal disease (IMD), after meningococcal serogroup B. In 2012, according to the European Centre for Disease Prevention and Control, 17% of IMD cases in Europe were caused by MenC (6). In addition, recent estimates from Spain (covering the period of 2005 to 2011), Germany (2002 to 2010), and Poland (2002 to 2011) found that MenC was responsible for 13.2%, 25.0%, and 36.6% of confirmed IMD cases, respectively (7–9).

The Hib-MenC-tetanus toxoid (TT) conjugate vaccine (Menitorix; GSK Vaccines) is a combination vaccine containing polyribosylribitol phosphate (PRP) from Hib and polysaccharide from MenC, each individually conjugated to TT as a carrier protein. The vaccine was developed to provide protection against Hib and MenC diseases, in a single injection, to infants and toddlers (10). Hib-MenC-TT offers an alternative vaccination schedule, compared with diphtheria-tetanus-acellular pertussis (DTPa)/Hib combined vaccines coadministered with MenC conjugate vaccines.

Hib-MenC-TT primary and booster vaccinations with different vaccination schedules and different combination vaccines were shown to be safe and immunogenic for infants and toddlers (11–27). Primary vaccination induced persistent antibodies against both antigens up to the second year of life (12–15). Persistence rates after booster vaccination varied in different studies (22, 23, 27), and there are no data available regarding antibody persistence after coadministration with a pneumococcal conjugate vaccine.

As mass vaccination schedules expand, there is a risk that adding more antigens to the schedule could lead to unexpected immune interferences (28, 29). If initial immune responses are not as robust as initially expected, then the antibody responses may not persist as long as protection is needed. The purpose of this extension study was to explore whether differences in initial antibody concentrations and titers for the coadministered antigens in the Hib-MenC and pneumococcal conjugate vaccines translated into differences in long-term persistence.

In the original randomized study, 1,548 healthy children were vaccinated with Hib-MenC-TT or a control MenC conjugate vaccine coadministered with a DTPa- or DTPa/Hib-containing vaccine and a pneumococcal conjugate vaccine. After primary (at 2, 4, and 6 months of age) and booster (at 11 to 18 months of age) vaccinations, immune responses were induced against all vaccine components (16, 17). In the current follow-up study, we evaluated the persistence of the immunogenicity of the Hib-MenC-TT conjugate vaccine up to 5 years after booster vaccination, with the evaluation of MenC antibody persistence as the primary objective. The persistence of antibodies to the pneumococcal conjugate vaccines administered in the study will be addressed in a separate publication.

## MATERIALS AND METHODS

### Ethical statement.

The antibody persistence study (ClinicalTrials.gov registration no. NCT00891176) was conducted in Germany, Poland, and Spain between May and December 2009 (year 2), May and November 2010 (year 3), and April and November 2012 (year 5). The protocol was approved by the following ethics committees: Comité Ético de Investigación Clínica del Hospital Universitario de Móstoles (Madrid, Spain), Comité Ético de Investigación Clínica del Hospital Clínico San Carlos (Madrid, Spain), Komisja Bioetyczna przy Okregowej Izbie Lekarskiej (Cracow, Poland), Ethik-Kommission der Bayerischen Landesarztekammer (Munich, Germany), Ethik-Kommission der Landesarztekammer Baden-Wurttemberg (Stuttgart, Germany), and Landesamt für Gesundheit und Soziales Geschaftss der Ethik-Kommission des Landes (Berlin, Germany). The study was conducted in accordance with the principles of good clinical practice and the Declaration of Helsinki, although some deviations in terms of study documentation and adherence to the study protocol were identified. Some additional good clinical practice deviations were noted at one of the study centers, which included the finding that annual reports and updates of protocol amendments and administrative changes had not been sent to regulatory authorities, as required by local law. After full investigation by an independent department and discussions with local regulatory agencies, however, it was determined that these findings did not have an impact on the safety of participants or on data integrity.

### Study design and participants.

Written informed consent was obtained from each child's parent or guardian. Healthy children who had completed primary and booster vaccinations in a previous study (ClinicalTrials.gov registration no. NCT00334334/00463437) (17) and were in the blood sampling subset in the booster study and whose parents or legal guardians were considered by the investigators to be able to comply with study requirements were included in this open, long-term persistence study. Children were excluded from the persistence study if they had received a MenC, Hib, hepatitis B virus (HBV), or pneumococcal vaccine or had developed MenC, Hib, hepatitis B, or invasive pneumococcal disease since the booster vaccination or had received an immune-modifying drug in the previous 6 months, a blood product in the previous 3 months, or an investigational drug or vaccine in the 30 days before blood sampling. In the previous primary-booster vaccination study, exclusion criteria included vaccination against diphtheria, tetanus, pertussis, polio, MenC, Hib, hepatitis B, or Streptococcus pneumoniae. Immunization with vaccines (such as hepatitis B vaccine) when the first dose was given within the first 2 weeks of life was permitted, in accordance with national recommendations.

In the initial phase III, open, randomized, multicenter study (17), Hib-MenC-TT or a control MenC conjugate vaccine (MenC-cross-reacting material 197 [CRM_197_] [Meningitec; Pfizer Inc.] or MenC-TT [NeisVac-C; Baxter Healthcare SA]) was coadministered with a DTPa- or DTPa/Hib-containing vaccine (all manufactured by GSK Vaccines) and one of two pneumococcal conjugate vaccines, i.e., a 10-valent pneumococcal nontypeable Haemophilus influenzae protein D conjugate vaccine (PHiD-CV) (Synflorix; GSK Vaccines) or a 7-valent pneumococcal CRM_197_ conjugate vaccine (7vCRM) (Prevenar/Prevnar; Pfizer Inc.). Hib-MenC-TT, pneumococcal conjugate vaccines, and DTPa-containing vaccines were administered at 2, 4, and 6 months of age, with booster vaccination at 11 to 18 months of age (17). The MenC-TT and MenC-CRM_197_ vaccines were administered at 2 and 4 months of age (in Poland, to comply with national recommendations, a third dose was offered at approximately 7 months of age) and at 11 to 18 months of age.

There were four study groups: children were randomized (1:1:1:1) to receive Hib-MenC-TT plus either PHiD-CV (Hib-MenC + PHiD-CV group) or 7vCRM (Hib-MenC + 7vCRM group), MenC-CRM_197_ plus PHiD-CV (MenC-CRM group), or MenC-TT plus PHiD-CV (MenC-TT group) (Table 1). In Germany and Poland, groups administered Hib-MenC-TT were given DTPa-HBV-inactivated polio virus (IPV) vaccine (Infanrix penta/Pediarix; GSK Vaccines) and groups administered MenC conjugate vaccines were given DTPa-HBV-IPV/Hib (Infanrix hexa; GSK Vaccines) as booster vaccination. Because hepatitis B booster vaccination is not recommended in Spain, the Spanish Hib-MenC-TT groups received DTPa-IPV (Infanrix-IPV; GSK Vaccines) while the control groups received DTPa-IPV/Hib (Infanrix-IPV/Hib; GSK Vaccines) as booster vaccination. In Poland, children received a single dose of hepatitis B vaccine at birth, according to national recommendations.

**TABLE 1:** Study groups^*a*^

Group	MenC vaccine	Pneumococcal vaccine	Coadministered vaccine
Hib-MenC + PHiD-CV	Hib-MenC-TT	PHiD-CV	DTPa-HBV-IPV or DTPa-IPV^*b*^
Hib-MenC + 7vCRM	Hib-MenC-TT	7vCRM	DTPa-HBV-IPV or DTPa-IPV^*b*^
MenC-CRM^*c*^	MenC-CRM_197_	PHiD-CV	DTPa-HBV-IPV/Hib or DTPa-IPV/Hib^*b*^
MenC-TT^*c*^	MenC-TT	PHiD-CV	DTPa-HBV-IPV/Hib or DTPa-IPV/Hib^*b*^

a The primary vaccination phase included doses at 2, 4, and 6 months. In the booster phase, vaccines were administered at 11 to 18 months.

b Because hepatitis B booster vaccination is not recommended in Spain, the Spanish Hib-MenC-TT groups received DTPa-IPV and the control groups received DTPa-IPV/Hib as booster vaccination.

c MenC-CRM_197_ and MenC-TT vaccines were administered at 2 and 4 months of age; in Poland, to comply with national recommendations, children were offered a third dose of MenC vaccines at ∼7 months of age.

### Study objectives.

The primary objective of this follow-up study was to evaluate the MenC antibody persistence after vaccination in all treatment groups that had received the Hib-MenC-TT conjugate vaccine, in terms of percentages of children with serum antibody levels above the assay cutoff value up to 60 months after booster vaccination (72 to 76 months of age). In this report, antibody persistence results are presented for blood samples obtained from children at approximately 3, 4, and 6 years of age. The secondary objectives included the evaluation of antibody persistence with respect to Hib capsular polysaccharide (anti-polyribosylribitol phosphate [PRP]) and hepatitis B surface antigen (anti-HBs) antibody concentrations.

### Immunogenicity assessments.

Blood samples of approximately 5 ml were collected at 36 to 40 months of age (i.e., approximately 24 months after booster vaccination), 48 to 52 months of age (i.e., approximately 36 months after booster vaccination), and 72 to 76 months of age (i.e., approximately 60 months after booster vaccination). Antibodies against MenC at year 5 were measured by the serum bactericidal antibody (SBA) assay performed at Public Health England (PHE), using rabbit complement (i.e., rabbit SBA [rSBA]), with a cutoff value of 1:8 as an indication of protection (30). Earlier time points were tested with an in-house rSBA-MenC assay performed at GSK Vaccines (16), which prevents direct comparison of the year 5 results with the initial results from earlier study time points (17). Anti-PRP antibodies were assessed with an in-house enzyme-linked immunosorbent assay (ELISA) performed at GSK Vaccines, for which concentrations of ≥0.15 μg/ml and ≥1 μg/ml are considered indicative of short- and long-term protection, respectively (31, 32). Antibodies against hepatitis B were assessed at GSK Vaccines using a commercial chemiluminescence immunoassay (Centaur; Siemens Healthcare Diagnostics). Anti-HBs seroprotection was defined as an antibody concentration above 10 mIU/ml (33).

### Safety assessments.

Serious adverse events (SAEs) for the primary and booster phases of this study were reported earlier (34). In this study, SAEs that were assessed by the investigators as being potentially vaccine or study procedure related or due to a lack of vaccine efficacy were documented retrospectively, following booster vaccination, and were recorded at every visit/contact during the study. According to the definition, an SAE was any untoward medical occurrence that resulted in death, was life-threatening, required hospitalization, or resulted in disability/incapacity. Although it was unlikely that SAEs related to vaccination would occur so late after vaccination, this study was designed to ensure that any event that manifested only long after vaccination and that the investigator thought could have been related to the vaccination or any event that was related to a lack of vaccine efficacy during this long-term persistence phase and that fulfilled the definition of an SAE would be recorded.

### Statistical analyses.

Results are presented for the according-to-protocol cohorts for antibody persistence after booster vaccination, which included all eligible children who received the full primary and booster vaccination courses corresponding to their group, were compliant with the study procedures, and had available assay results. For each group, the antibody geometric mean concentrations (GMCs), the antibody geometric mean titers (GMTs), and the percentages of participants retaining antibody GMCs or GMTs above the predefined thresholds were calculated, with associated 95% confidence intervals (CIs).

Exploratory statistical analyses were performed in which potential differences between groups were defined when the asymptotic standardized 95% CI for the difference between the two groups in percentages of children with titers or concentrations above the proposed cutoff values did not contain the value 0 or when the 95% CI for the GMT/GMC ratios between groups did not contain the value 1. This analysis was performed using a one-way analysis of variance model based on the log_10_ transformation of the titers or concentrations, using the vaccine group and country as the only covariates. The results of all exploratory group comparisons should be interpreted with caution considering that there was no adjustment for multiplicity for these comparisons and the clinical relevance of the differences was not prespecified. The statistical analyses were performed using SAS software (version 9.22 on Windows; SAS Institute, Cary, NC, USA).

## RESULTS

### Study participants.

**FIG 1:**
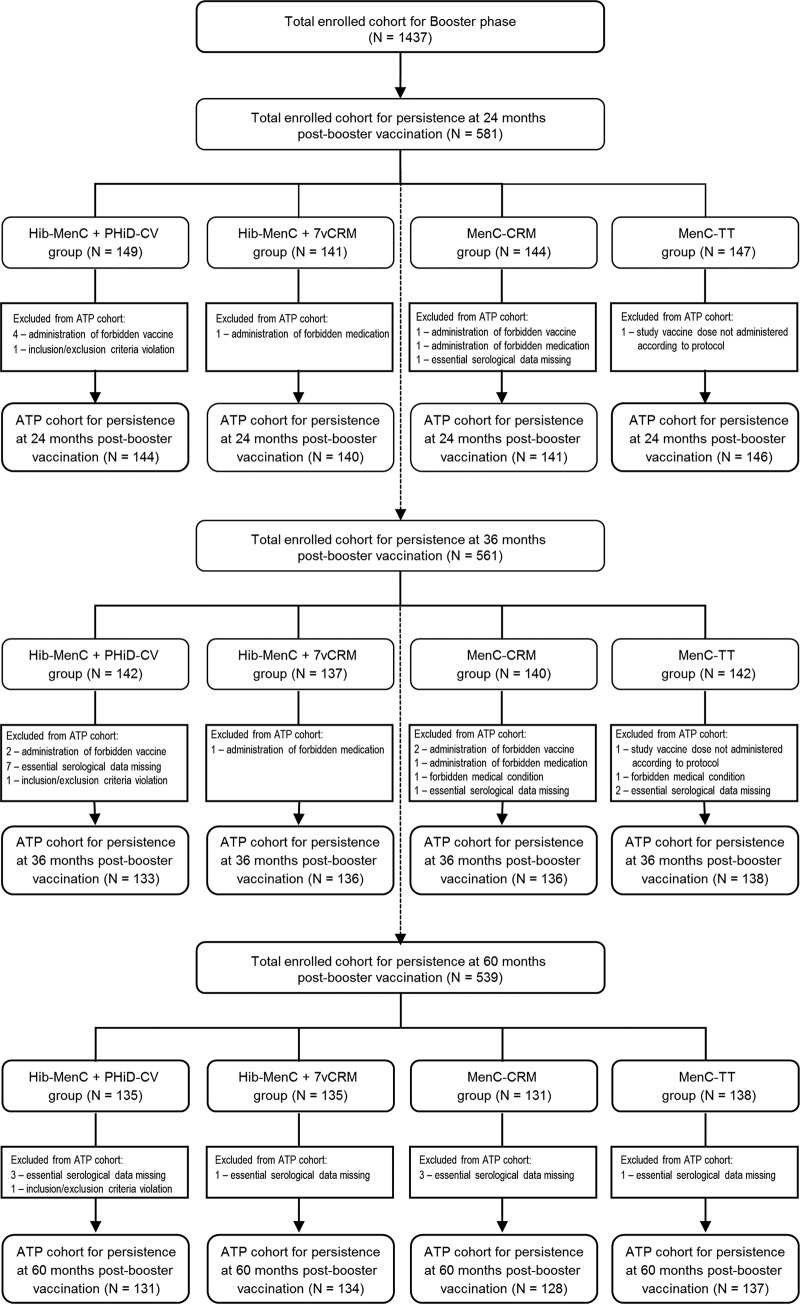
Disposition of study children and reasons for exclusion from according-to-protocol (ATP) cohorts for persistence at 24, 36, and 60 months after booster vaccination. N, number of study participants for the specified group.

Of the 1,437 children who were part of the total enrolled cohort for the booster phase, 581, 561, and 539 were included in the total enrolled cohorts for persistence at 24, 36, and 60 months after booster vaccination, respectively. The according-to-protocol cohorts for antibody persistence included 571 children at 24 months after booster vaccination, 543 children at 36 months after booster vaccination, and 530 children at 60 months after booster vaccination. The reasons for exclusion from the according-to-protocol cohorts and the distributions for the study groups are presented in Fig. 1.

Across the four vaccine groups, the mean ages of the children in the according-to-protocol cohorts for persistence were similar between groups and the majority of the children were of European descent (Table 2). The proportions of male and female subjects were similar across all groups, with a larger proportion of male subjects in the MenC-TT group and a larger proportion of female subjects in the Hib-MenC + PHiD-CV group. The gender and racial distributions in the according-to-protocol cohorts for persistence were consistent with the primary study (17).

**TABLE 2:** Demographic characteristics of the study children (according-to-protocol cohorts for antibody persistence at 24 months [year 2], 36 months [year 3], and 60 months [year 5] after booster vaccination)

Parameter and time point	Hib-MenC + PHiD-CV		Hib-MenC + 7vCRM		MenC-CRM		MenC-TT	
	*n*^*a*^	Value	*n*	Value	*n*	Value	*n*	Value
Age (mean ± SD) (mo)								
Year 2	144	37.3 ± 1.2	140	37.3 ± 1.3	141	37.1 ± 1.2	146	37.2 ± 1.2
Year 3	133	49.2 ± 1.5	136	48.7 ± 1.2	136	48.8 ± 1.3	138	48.7 ± 1.1
Year 5	131	72.9 ± 1.0	134	72.9 ± 1.2	128	72.8 ± 1.0	137	72.9 ± 1.1
Female (%)								
Year 2	144	57.6	140	50.7	141	50.4	146	44.5
Year 3	133	60.2	136	52.2	136	50.7	138	43.5
Year 5	131	58.8	134	50.7	128	49.2	137	46.0
European descent (%)								
Year 2	144	95.1	140	100	141	95.7	146	98.6
Year 3	133	95.5	136	99.3	136	96.3	138	97.8
Year 5	131	96.2	134	99.3	128	96.9	137	97.8

a *n*, total number of children in the group.

### Immunogenicity against MenC.

Approximately 5 years after booster vaccination, the percentages of children with rSBA-MenC titers of ≥8, as measured by the PHE assay, were between 24.2% and 40.1% in all groups, with the highest value being observed in the MenC-TT group (Table 3). GMTs ranged from 7.2 in the MenC-CRM group to 11.9 in the MenC-TT group. At year 3, rSBA-MenC titers of ≥8, as measured by the GSK Vaccines assay, were retained by at least 72.4% of children in each group (see Table S1 in the supplemental material).

**TABLE 3:** Serum bactericidal activity against MenC approximately 5 years after booster vaccination (according-to-protocol cohorts for antibody persistence at 60 months after booster vaccination)

Group	*n*^*a*^	% with rSBA-MenC titers of ≥8 (95% CI)	rSBA-MenC GMT (95% CI)
Hib-MenC + PHiD-CV	130	38.5 (30.1–47.4)	10.1 (7.9–12.9)
Hib-MenC + 7vCRM	134	25.4 (18.3–33.6)	8.5 (6.6–11.1)
MenC-CRM	128	24.2 (17.1–32.6)	7.2 (5.8–8.9)
MenC-TT	137	40.1 (31.9–48.9)	11.9 (8.9–16.0)

a *n*, total number of children with available results.

Based on exploratory analyses, the percentages of children with rSBA-MenC titers of ≥8 at year 5 were higher in the MenC-TT and Hib-MenC + PHiD-CV groups than in the MenC-CRM group and in the MenC-TT group than in the Hib-MenC + 7vCRM group (Table 4). The rSBA-MenC GMT was higher in the MenC-TT group than in the MenC-CRM group. No difference between the MenC-TT and Hib-MenC + PHiD-CV groups was observed. Similar observations were made at year 3 (see Table S2 in the supplemental material).

**TABLE 4:** Differences between groups (first group minus second group) in percentages of children with rSBA-MenC titers above the threshold and rSBA-MenC GMT ratios (first group to second group) approximately 5 years after booster vaccination (exploratory analyses; according-to-protocol cohorts for antibody persistence at 60 months after booster vaccination)^*a*^

Group comparison	Difference in proportions of children with rSBA-MenC titers of ≥8 (95% CI)	rSBA-MenC GMT ratio (95% CI)
MenC-CRM vs MenC-TT	**−15.93 (−26.79 to −4.67)**	**0.68 (0.47–0.96)**
MenC-CRM vs Hib-MenC + PHiD-CV	**−14.24 (−25.26 to −2.92)**	0.73 (0.53–1.01)
MenC-CRM vs Hib-MenC + 7vCRM	−1.15 (−11.62 to 9.39)	0.92 (0.67–1.28)
MenC-TT vs Hib-MenC + PHiD-CV	1.68 (−10.02 to 13.32)	1.15 (0.79–1.68)
MenC-TT vs Hib-MenC + 7vCRM	**14.77 (3.59 to 25.62)**	1.38 (0.95–2.01)

a For differences, 95% CIs not including 0 were regarded as indicating significant potential differences between groups. For GMT ratios, 95% CIs not including 1 indicated significant potential differences. Bold type indicates comparisons for which the exploratory analysis suggests a potentially significant difference.

### Immunogenicity against Hib PRP.

Approximately 5 years after booster vaccination, the percentages of children with anti-PRP concentrations of ≥0.15 μg/ml remained high in all groups (≥98.5%) (Table 5). Among all groups, at least 57.5% of children had anti-PRP concentrations of ≥1 μg/ml, with the highest percentages among the Hib-MenC-TT recipients (Hib-MenC + PHiD-CV and Hib-MenC + 7vCRM groups). Anti-PRP GMCs at year 5 ranged from 1.65 in the MenC-TT group to 2.95 in the Hib-MenC + PHiD-CV group.

**TABLE 5:** Anti-PRP antibody persistence (according-to-protocol cohorts for antibody persistence at 60 months after booster vaccination)

Group and time point^*a*^	*n*^*b*^	% with anti-PRP antibody levels of ≥0.15 μg/ml (95% CI)	% with anti-PRP antibody levels of ≥1 μg/ml (95% CI)	Anti-PRP antibody GMC (95% CI) (μg/ml)
Hib-MenC + PHiD-CV				
Postprimary	130	98.5 (94.6–99.8)	97.7 (93.4–99.5)	13.71 (11.11–16.93)
Postbooster M1	130	100 (97.2–100)	100 (97.2–100)	90.53 (75.73–108.23)
Postbooster M24	121	100 (97.0–100)	93.4 (87.4–97.1)	4.21 (3.41–5.21)
Postbooster M36	122	100 (97.0–100)	90.2 (83.4–94.8)	3.76 (3.03–4.66)
Postbooster M60	130	100 (97.2–100)	84.6 (77.2–90.3)	2.95 (2.40–3.62)
Hib-MenC + 7vCRM				
Postprimary	132	100 (97.2–100)	97.0 (92.4–99.2)	10.70 (8.87–12.90)
Postbooster M1	130	100 (97.2–100)	100 (97.2–100)	65.65 (52.76–81.69)
Postbooster M24	125	100 (97.1–100)	84.8 (77.3–90.6)	3.77 (3.04–4.69)
Postbooster M36	125	99.2 (95.6–100)	79.2 (71.0–85.9)	2.80 (2.27–3.46)
Postbooster M60	132	100 (97.2–100)	75.8 (67.5–82.8)	2.56 (2.07–3.17)
MenC-CRM				
Postprimary	127	98.4 (94.4–99.8)	86.6 (79.4–92.0)	4.23 (3.36–5.32)
Postbooster M1	127	100 (97.1–100)	99.2 (95.7–100)	33.49 (27.11–41.37)
Postbooster M24	118	100 (96.9–100)	78.0 (69.4–85.1)	2.51 (1.99–3.16)
Postbooster M36	123	99.2 (95.6–100)	67.5 (58.4–75.6)	1.94 (1.56–2.41)
Postbooster M60	127	99.2 (95.7–100)	66.9 (58.0–75.0)	1.66 (1.34–2.05)
MenC-TT				
Postprimary	135	100 (97.3–100)	95.6 (90.6–98.4)	6.48 (5.48–7.67)
Postbooster M1	134	100 (97.3–100)	100 (97.3–100)	36.35 (30.22–43.73)
Postbooster M24	130	100 (97.2–100)	77.7 (69.6–84.5)	2.29 (1.84–2.85)
Postbooster M36	128	99.2 (95.7–100)	62.5 (53.5–70.9)	1.78 (1.42–2.24)
Postbooster M60	134	98.5 (94.7–99.8)	57.5 (48.6–66.0)	1.65 (1.30–2.09)

a Postprimary, 1 month after the third dose at 6 months of age; postbooster M1, 1 month after booster vaccination; postbooster M24, approximately 24 months after booster vaccination; postbooster M36, approximately 36 months after booster vaccination; postbooster M60, approximately 60 months after booster vaccination.

b *n*, total number of children with available results.

Based on exploratory analyses, there was no significant potential difference between groups in percentages of children achieving anti-PRP antibody concentrations of ≥0.15 μg/ml. Anti-PRP antibody GMCs were lower for the control MenC vaccine recipients than the Hib-MenC-TT groups, regardless of MenC vaccine type, and there was no difference in anti-PRP antibody GMCs between the two MenC vaccine groups (Table 6).

**TABLE 6:** Differences between groups (first group minus second group) in percentages of children with anti-PRP antibody concentrations above the threshold and anti-PRP GMC ratios (first group to second group) approximately 5 years after booster vaccination (exploratory analyses; according-to-protocol cohorts for antibody persistence at 60 months after booster vaccination)^*a*^

Group comparison	Difference in proportions of children with anti-PRP antibody levels of ≥0.15 μg/ml (95% CI)	Difference in proportions of children with anti-PRP antibody levels of ≥1 μg/ml (95% CI)	Anti-PRP antibody GMC ratio (95% CI)
MenC-CRM vs MenC-TT	0.71 (−2.97 to 4.59)	9.47 (−2.34 to 20.99)	0.96 (0.70–1.31)
MenC-CRM vs Hib-MenC + PHiD-CV	−0.79 (−4.34 to 2.11)	**−17.69 (−27.93 to −7.31)**	**0.54 (0.41–0.73)**
MenC-CRM vs Hib-MenC + 7vCRM	−0.79 (−4.34 to 2.06)	−8.83 (−19.76 to 2.20)	**0.64 (0.47–0.87)**
MenC-TT vs Hib-MenC + PHiD-CV	−1.49 (−5.29 to 1.41)	**−27.15 (−37.34 to −16.49)**	**0.56 (0.41–0.75)**
MenC-TT vs Hib-MenC + 7vCRM	−1.49 (−5.29 to 1.37)	**−18.29 (−29.18 to −6.99)**	**0.65 (0.47–0.89)**

a For differences, 95% CIs not including 0 were regarded as indicating significant potential differences between groups. For GMC ratios, 95% CIs not including 1 indicated significant potential differences. Bold type indicates comparisons for which the exploratory analysis suggests a potentially significant difference.

### Immunogenicity against hepatitis B.

Considering the anti-HBs results determined with the chemiluminescence assay, at least 72.8% of children had antibody concentrations of ≥10 mIU/ml approximately 60 months after booster vaccination (Table 7). Anti-HBs antibody GMCs declined by 60 months after booster vaccination and were between 45.7 and 85.4 mIU/ml at 60 months after booster vaccination.

**TABLE 7:** Anti-HBs antibody persistence at year 5 after booster vaccination, as assessed with a chemiluminescence assay (according-to-protocol cohorts for antibody persistence at 60 months after booster vaccination)^*a*^

Group and time point^*b*^	*n*^*c*^	% with anti-HBs antibody levels of ≥10 mIU/ml (95% CI)	Anti-HBs antibody GMC (95% CI) (mIU/ml)
Hib-MenC + PHiD-CV			
Postbooster M24	122	88.5 (81.5–93.6)	154.5 (106.8–223.5)
Postbooster M36	118	84.7 (77.0–90.7)	92.3 (63.8–133.6)
Postbooster M60	125	72.8 (64.1–80.4)	45.7 (32.2–64.8)
Hib-MenC + 7vCRM			
Postbooster M24	119	93.3 (87.2–97.1)	218.9 (152.4–314.4)
Postbooster M36	119	89.9 (83.0–94.7)	142.5 (100.3–202.6)
Postbooster M60	128	84.4 (76.9–90.2)	85.4 (60.4–120.6)
MenC-CRM			
Postbooster M24	118	92.4 (86.0–96.5)	211.1 (147.1–303.1)
Postbooster M36	120	86.7 (79.3–92.2)	130.8 (91.9–186.2)
Postbooster M60	122	82.0 (74.0–88.3)	66.7 (47.2–94.1)
MenC-TT			
Postbooster M24	130	90.8 (84.4–95.1)	189.2 (134.2–266.7)
Postbooster M36	127	88.2 (81.3–93.2)	128.4 (91.3–180.6)
Postbooster M60	129	79.8 (71.9–86.4)	64.7 (46.4–90.2)

a A booster vaccination for HBV was not given in Spain, in accordance with national recommendations. Data shown here include subjects from all countries combined.

b Postbooster M24, approximately 24 months after booster vaccination; postbooster M36, approximately 36 months after booster vaccination; postbooster M60, approximately 60 months after booster vaccination.

c *n*, total number of children with available results.

### Safety.

No SAEs considered by the investigators to be potentially vaccine or study procedure related or due to a lack of vaccine efficacy were reported following administration of the booster vaccination.

## DISCUSSION

This study reports the antibody persistence in healthy children up to 5 years after the Hib and MenC full vaccination course. At the time the study was conducted, children in the United Kingdom used to receive concomitant doses of Hib-MenC and pneumococcal conjugate vaccines as primary vaccinations. Our data are important in comparing persistence after booster vaccination, since children in the United Kingdom currently receive booster doses of Hib-MenC and pneumococcal conjugate vaccines simultaneously at 12 to 13 months of age (35).

For MenC, the percentages of children retaining seroprotective rSBA-MenC titers at year 5 after booster vaccination were 24.2%, 25.4%, 38.5%, and 40.1% in the MenC-CRM, Hib-MenC + 7vCRM, Hib-MenC + PHiD-CV, and MenC-TT groups, respectively. In contrast, retention of anti-PRP antibodies was nearly universal in all groups; at least 98.5% of children in each group were observed to have anti-PRP concentrations of ≥0.15 μg/ml at 5 years after booster vaccination.

The percentages of children with seroprotective rSBA-MenC titers of ≥8 were considerably lower than the rates of 72.4% to 96.4% reported 3 years after booster vaccination. The assays were performed in different laboratories with different procedures, however, and the results cannot be directly compared. It has been observed that the GSK Vaccines rSBA-MenC assay used previously has a higher sensitivity than the PHE rSBA-MenC assay used in the current follow-up study, especially for naturally acquired antibodies (36). In addition, the PHE laboratory uses a discontinuous method to interpolate titers from the standard curve, whereas GSK Vaccines uses a continuous method. The discontinuous method of titer calculation is known to result in lower titers than the continuous method.

The proportions of children with seroprotective rSBA-MenC titers of ≥8 in our study were consistent with the rates of 22% to 43% (measured with the same rSBA assay at PHE) reported in a previous study in the United Kingdom at 24 months after the administration of a Hib-MenC-TT booster dose to toddlers (12 to 14 months of age) (19). In that study, infants had been primed with two doses of MenC-CRM_197_ or MenC-TT at 2 and 3 or 2 and 4 months of age (19). In contrast, in a study in Spain in which the GSK Vaccines rSBA-MenC assay was used, greater percentages were observed more than 5 years after Hib-MenC-TT booster vaccination in toddlers, following Hib-MenC-TT or MenC-TT priming in infancy on a 2/4/6-month schedule (78% to 97%) (23). However, these results cannot be directly compared with the current study results, as different rSBA assay procedures were used.

The low MenC titers at 5 years after vaccination suggested that individuals may no longer be protected or contribute to herd immunity. The introduction of a MenC adolescent booster dose at ∼14 years of age was adopted in the United Kingdom in 2013, based on advice from the Joint Committee on Vaccination and Immunisation (37). The adolescent booster vaccination at 12 years of age was also incorporated into the vaccination calendar of all autonomous Spanish regions in 2014 (7).

The exploratory analyses revealed possible differences between groups in MenC antibody persistence. The percentages of children with rSBA-MenC titers of ≥8 at year 5 after booster vaccination were higher in the MenC-TT and Hib-MenC + PHiD-CV groups than in the MenC-CRM group and in the MenC-TT group than in the Hib-MenC + 7vCRM group. MenC GMTs were observed to be higher in the MenC-TT group than in the MenC-CRM group, which is consistent with previous observations suggesting that TT is a better carrier protein for MenC priming than is CRM_197_, in terms of seroprotection and GMTs (19, 38).

A clear effect of the coadministered pneumococcal conjugate vaccines could not be observed, since there were no significant potential differences in terms of MenC GMTs between PHiD-CV and 7vCRM recipients. Moreover, the control vaccines MenC-CRM_197_ and MenC-TT were coadministered only with PHiD-CV; no groups receiving these control MenC vaccines together with 7vCRM were included in this study.

High rates of seroprotection against the Hib antigen were observed in all groups. There was no significant potential difference between groups in the percentages of children with anti-PRP antibody concentrations of ≥0.15 μg/ml, and there was no decline in comparison with earlier time points. Comparison of anti-PRP antibody GMCs between groups showed better long-term persistence 5 years after booster vaccination for the Hib-MenC-TT recipients than for the DTPa/Hib recipients, regardless of the coadministered pneumococcal conjugate vaccine, which is consistent with a previous report (23). There was only a slight decrease in the year 5 anti-PRP antibody GMC values, compared to the previous time points (up to 3 years after booster vaccination). The high level of anti-PRP antibody persistence in all groups suggests that an additional Hib booster dose is not needed.

Infants and toddlers are routinely vaccinated against hepatitis B in many countries, with the expectation that such vaccinations will protect against infections that may be acquired years in the future. The purpose of assessing the hepatitis B antibody persistence was to explore the percentages of subjects retaining seroprotective antibody concentrations 5 years after vaccination with the various regimens. Our results showed that the anti-HBs seroprotection rates 5 years after booster vaccination ranged from 73% to 84% in all groups. These percentages appeared to be smaller than those at earlier time points (2 and 3 years after booster vaccination). Statistical comparisons were not performed, however, and the anti-HBs data from our study must be interpreted with caution.

In conclusion, 5 years after booster vaccination, protective anti-PRP antibody concentrations persisted in at least 98.5% of children in all groups, but protective rSBA-MenC antibody titers were shown to persist in only 24.2 to 40.1% of children, with the highest values in the Hib-MenC + PHiD-CV and MenC-TT groups. These results are consistent with previously reported Hib-MenC-TT vaccine data. No SAEs considered possibly related to vaccination were reported during the 5-year persistence phase of the study.

## Supplementary Material

Supplemental material
