# Supplementary material for: Antibody Persistence in Young Children 5 Years after Vaccination with a Combined Haemophilus influenzae Type b-Neisseria meningitidis Serogroup C Conjugate Vaccine Coadministered with Diphtheria-Tetanus-Acellular Pertussis-Based and Pneumococcal Conjugate Vaccines
Source: Clin Vaccine Immunol. 2016 Jul 5;23(7):555–63. doi: 10.1128/CVI.00057-16 (PMC4933777; doi:10.1128/CVI.00057-16)
Supplement: Supplemental material [file supp_23_7_555__index.html]

Supplemental material 

# Antibody Persistence in Young Children 5 Years after Vaccination with a Combined Haemophilus influenzae Type b-Neisseria meningitidis Serogroup C Conjugate Vaccine Coadministered with Diphtheria-Tetanus-Acellular Pertussis-Based and Pneumococcal Conjugate Vaccines

## Supplemental material

- Supplemental file 1 -

  Table S1. rSBA-MenC antibody persistence. Table S2. Differences between groups in percentages of children with rSBA-MenC titers above the threshold and rSBA-MenC GMT ratios approximately 3 years after booster vaccination.

  PDF, 146K
