# Supplementary material for: Antibody Persistence in Young Children 5 Years after Vaccination with a Combined Haemophilus influenzae Type b-Neisseria meningitidis Serogroup C Conjugate Vaccine Coadministered with Diphtheria-Tetanus-Acellular Pertussis-Based and Pneumococcal Conjugate Vaccines
Source: Clin Vaccine Immunol. 2016 Jul 5;23(7):555–63. doi: 10.1128/CVI.00057-16 (PMC4933777; doi:10.1128/CVI.00057-16)
Supplement: Supplemental material [file CVI.00057-16_zcd999095363so1.pdf]

Group	Time point	rSBA-MenC (95% CI)		
		N	% ≥8	GMT
Hib-MenC + PHiD-CV	Post-primary dose 2	130	97.7 (93.4–99.5)	507.2 (402.7–638.8)
	Post-primary dose 3	106	100 (96.6–100)	1590.7 (1278.1–1979.8)
	Post-booster: M1	63	100 (94.3–100)	5393.7 (4092.2–7109.1)
	Post-booster: M24	130	88.5 (81.7–93.4)	162.9 (120.5–220.3)
	Post-booster: M36	128	84.4 (76.9–90.2)	112.4 (81.8–154.6)
Hib-MenC + 7vCRM	Post-primary dose 2	127	97.6 (93.3–99.5)	454.1 (360.9–571.4)
	Post-primary dose 3	96	99.0 (94.3–100)	1164.5 (902.3–1502.9)
	Post-booster: M1	66	100 (94.6–100)	2978.1 (2202.5–4026.9)
	Post-booster: M24	132	82.6 (75.0–88.6)	87.2 (63.5–119.9)
	Post-booster: M36	128	76.6 (68.3–83.6)	68.1 (48.4–95.9)
MenC-CRM	Post-primary dose 2	131	99.2 (95.8–100)	1284.1 (1059.7–1556.1)
	Post-booster: M1	80	100 (95.5–100)	2687.7 (2087.9–3459.9)
	Post-booster: M24	132	86.4 (79.3–91.7)	96.0 (71.9–128.2)
	Post-booster: M36	134	72.4 (64.0–79.8)	45.3 (33.2–61.8)
MenC-TT	Post-primary dose 2	137	100 (97.3–100)	1410.1 (1181.8–1682.5)
	Post-booster: M1	76	100 (95.3–100)	4566.5 (3669.8–5682.2)
	Post-booster: M24	136	99.3 (96.0–100)	242.0 (193.6–302.4)
	Post-booster: M36	137	96.4 (91.7–98.8)	146.8 (116.2–185.4)

Supplementary Table 1. rSBA-MenC antibody persistence (ATP cohort for antibody persistence at 36 months post-booster vaccination)

GMT, geometric mean titre; Post-primary dose 2, 2 months after second Hib-MenC-TT, MenC-CRM and MenC-TT doses at 4 months of age; Post-primary dose 3, 1 month after third Hib-MenC-TT dose at 6 months of age; Post-booster: M1, 1 month after booster vaccination; Post-booster: M24, approximately 24 months after booster vaccination; Post-booster: M36, approximately 36 months after booster vaccination.

Group comparison	rSBA-MenC (95% CI)	
	**Difference in % of children achieving ≥8**	**GMT ratio**
**MenC-CRM vs. MenC-TT**	**-23.96 (-32.52; -16.02)**	**0.30 (0.21; 0.45)**
**MenC-CRM vs. Hib-MenC + PHiD-CV**	**-11.99 (-21.85; -2.01)**	**0.40 (0.26; 0.62)**
**MenC-CRM vs. Hib-MenC + 7vCRM**	-4.17 (-14.70; 6.46)	0.68 (0.43; 1.06)
**MenC-TT vs. Hib-MenC + PHiD-CV**	**11.98 (5.21; 19.70)**	1.30 (0.88; 1.92)
**MenC-TT vs. Hib-MenC + 7vCRM**	**19.79 (12.12; 28.27)**	**2.15 (1.44; 3.21)**

Supplementary Table 2. Differences between groups (first group minus second group) in percentages of children with rSBA-MenC titres above the threshold and rSBA-MenC GMT ratios (first group over second group) approximately 3 years post-booster vaccination (exploratory analyses; according-to-protocol cohort for antibody persistence at 36 months post-booster vaccination)

rSBA, serum bactericidal assay using rabbit complement; 95% CIs of differences not including 0 were regarded as indicative that a significant potential difference might exist between groups. For GMT ratio, 95% CIs not including 1 indicated that a significant potential difference might exist. GMT = geometric mean titre; 95% CI, 95% confidence interval. **BOLD** = comparison for which the exploratory analysis suggests that a potentially significant difference may exist.
